# Biallelic variants in *CENPF* causing a phenotype distinct from Strømme syndrome

**DOI:** 10.1002/ajmg.c.31973

**Published:** 2022-04-30

**Authors:** Gerarda Cappuccio, Simona Brillante, Roberta Tammaro, Michele Pinelli, Margherita Lucia De Bernardi, Maria Grazia Gensini, Emilia K. Bijlsma, Tamara T. Koopmann, Mariette J. V. Hoffer, Kimberly McDonald, Laura G. Hendon, Sofia Douzgou, Charulata Deshpande, Stefano D'Arrigo, Annalaura Torella, Vincenzo Nigro, Brunella Franco, Nicola Brunetti‐Pierri

**Affiliations:** ^1^ Department of Translational Medicine, Section of Pediatrics Federico II University Naples Italy; ^2^ Telethon Institute of Genetics and Medicine Naples Italy; ^3^ Department of Clinical Genetics Leiden University Medical Center Leiden The Netherlands; ^4^ Department of Pediatrics University of Mississippi Medical Center Jackson Mississippi USA; ^5^ Department of Medical Genetics Haukeland University Hospital Bergen Norway; ^6^ Division of Evolution, Infection and Genomics, School of Biological Sciences University of Manchester Manchester UK; ^7^ Manchester Centre for Genomic Medicine St Mary's Hospital Manchester UK; ^8^ Department of Pediatric Neuroscience Fondazione IRCCS Istituto Neurologico C. Besta Milan Italy; ^9^ Department of Precision Medicine University of Campania ‘Luigi Vanvitelli’ Naples Italy; ^10^ Scuola Superiore Meridionale School for Advanced Studies Naples Italy

**Keywords:** anterior chamber defect, CENPF, cilia, duodenal atresia, Strømme syndrome

## Abstract

Biallelic loss‐of‐function (LoF) variants in *CENPF* gene are responsible for Strømme syndrome, a condition presenting with intestinal atresia, anterior ocular chamber anomalies, and microcephaly. Through an international collaboration, four individuals (three males and one female) carrying *CENPF* biallelic variants, including two missense variants in homozygous state and four LoF variants, were identified by exome sequencing. All individuals had variable degree of developmental delay/intellectual disability and microcephaly (ranging from −2.9 SDS to −5.6 SDS) and a recognizable pattern of dysmorphic facial features including inverted‐V shaped interrupted eyebrows, epicanthal fold, depressed nasal bridge, and pointed chin. Although one of the cases had duodenal atresia, all four individuals did not have the combination of internal organ malformations of Strømme syndrome (intestinal atresia and anterior eye segment abnormalities). Immunofluorescence analysis on skin fibroblasts on one of the four cases with the antibody for ARL13B that decorates primary cilia revealed shorter primary cilia that are consistent with a ciliary defect. This case‐series of individuals with biallelic *CENPF* variants suggests the spectrum of clinical manifestations of the disorder that may be related to *CENPF* variants is broad and can include phenotypes lacking the cardinal features of Strømme syndrome.

## 1 INTRODUCTION

CENPF is a microtubule‐regulating protein with a dual role as kinetochore protein in spindle orientation and ciliogenesis (Waters et al., 2015; Coppieters et al., 2010). To date, biallelic truncating variants in *CENPF* have been identified in eight patients and six fetuses with clinical features of Strømme syndrome (Alghamdi et al., 2020; Caridi et al., 2021; Filges et al., 2016; Ozkinay et al., 2017; Waters et al., 2015) but also in one child with nonsyndromic microcephaly and learning disability (Waters et al., 2015). Strømme syndrome is a condition characterized by the rare combination of apple peel intestinal atresia, anterior eye segment abnormalities, and cranial anomalies (mostly microcephaly; Filges et al., 2016). Additional reported findings include intellectual disability (ID), cerebral anomalies, congenital heart defects, renal abnormalities, and skeletal dysplasia. Craniofacial features may include epicanthal folds, large and low‐set ears, large mouth, micrognathia, and fine and sparse hairs. In some cases, the condition is lethal early in life, whereas other patients survived into adulthood with mild cognitive impairment (Alghamdi et al., 2020; Waters et al., 2015). All cases reported so far were found to harbor loss‐of‐function (LoF) variants in *CENPF*. We report four individuals with *CENPF* pathogenic variants, including two individuals with homozygous missense variants in *CENPF* that are reported for the first time. Moreover, these four cases differ from the recurrent pattern of abnormalities of Strømme syndrome, suggesting a broad spectrum of clinical manifestations for CENPF‐related disease.

## 2 CASE REPORTS

### 2.1 Proband 1

**FIGURE 1:**
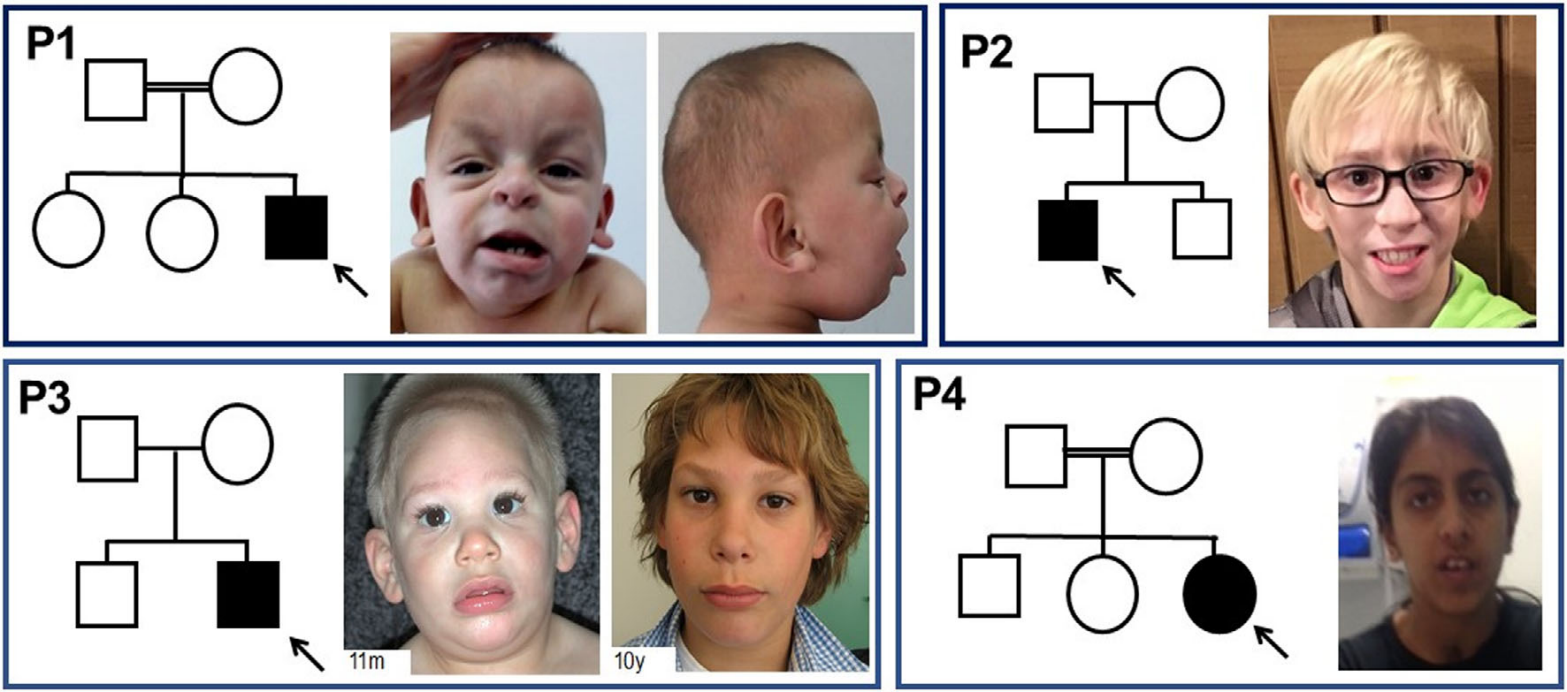
Pedigrees and facial features of the presented cases. Dysmorphic features include inverted‐V shaped interrupted eyebrows, epicanthal folds and peri‐orbital fullness, and large/prominent ears. m, months; y, years

**FIGURE 2:**
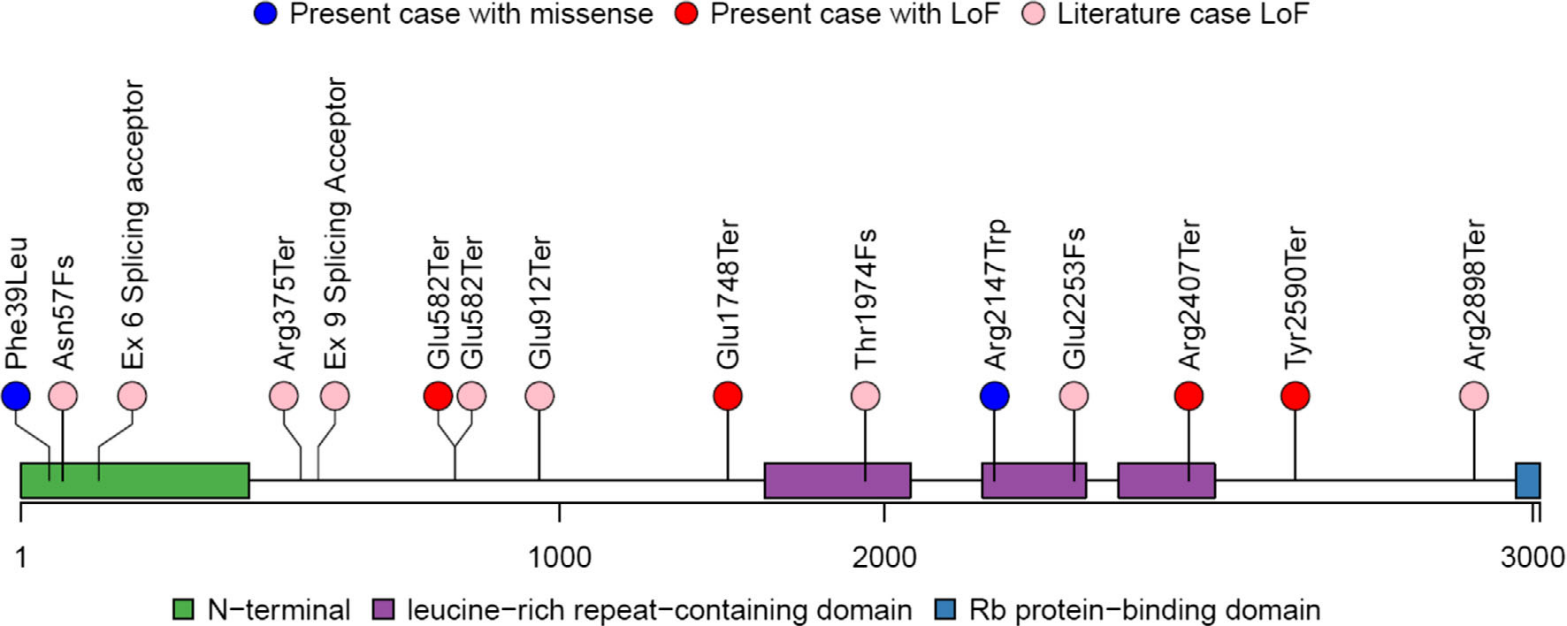
Location of *CENPF* variants. *CENPF* variants of the four patients herein described and previously reported cases are shown (NM_016343.3). *CENPF* missense variants (in blue) are reported herein for the first time

The proband is the third and last child of first‐cousin parents from Pakistani origin. His two older sisters were in good health. He was born at term of an unremarkable pregnancy with a birth weight of 2,720 g (third centile). At birth facial dysmorphisms were noted and on day two of life, he had respiratory distress requiring oxygen. He walked independently at 13 months of life and pronounced his first words at 18 months of age. At the age of 4 years 7 months, he could pronounce 20 words but no complete sentences, and he understood and followed simple commands. On clinical evaluation at 4 years and 7 months of age, his weight was 16 kg (19th centile), his stature was 105.5 cm (39th centile), and his occipito‐frontal circumference (OFC) was 48 cm (−3 SDS). He showed down‐slanting palpebral fissures, deep‐set eyes, flat philtrum, bulbous nasal tip, anteverted nares, low‐set ears, and shawl scrotum (Figure 1). An ophthalmological evaluation showed bilaterally excavation of the optic disk and the visual evoked potential were abnormal. No abnormalities of the anterior chamber were observed. On cardiac ultrasounds, atrial septal defect, and Ebstein‐like tricuspid valvular dysplasia were noted. Abdominal ultrasound showed unilateral mild renal pelvis dilatation. Array‐CGH did not identify any alterations. For the suspicion of Aarskog–Scott syndrome, sequencing of *FGD1* was performed but it did not reveal any pathogenic variants. The child was then enrolled in the Telethon Undiagnosed Program (TUDP) and trio genomic DNA underwent exome sequencing (ES) that revealed a homozygous variant in *CENPF* gene (NM_016343):c.117T>G:p.Phe39Leu, (hg19, chr1:214787214T>G; Figure 2) that was confirmed by Sanger sequencing. Each parent was found to be heterozygous for the variant and both unaffected sisters were also heterozygous. The variant was very rare in gnomAD (allele frequency 0.00003986) but was not found in homozygous state. The variant was predicted to be pathogenic and likely pathogenic by SIFT and Polyphen, respectively, with a CADD score of 24.7. The variant is classified as variant of unknown significance according to ACMG criteria (PM2, PM3, and PP3; Richards et al., 2015). Targeted analysis of exome data for variants in cilia genes failed to detect other candidate variants besides those identified in *CENPF*.

### 2.2 Proband 2

The proband is the first child of nonconsanguineous Caucasian parents. He has a healthy younger brother and an unremarkable family history. He was born after 37 weeks of gestation by vaginal delivery with a birth weight of 2381 g (−2.7 SDS). He had increased nuchal translucency on prenatal ultrasound, and at birth had lymphedema of the feet. The lymphedema resolved but returned several years later. After birth, he was admitted for feeding difficulties and multiple congenital anomalies, which included coarctation of the aorta, bicuspid aortic valve, duodenal atresia, horseshoe kidney, intestinal malrotation, and microcephaly. Other features included short stature, failure to thrive, bilateral mild conductive hearing loss, ocular anomalies (unilateral posterior subcapsular polar cataract and esotropia), vesicoureteral reflux, and cryptorchidism. He developed significant gastrointestinal complications, with recurrent episodes of acute pancreatitis associated with pancreatic stones, and multiple episodes of gastrointestinal bleeding due to duodenal and gastric varices. He also had chronic daily headaches, and a brain MRI at 11 years showed mild decrease in size of the frontal lobes with possible malformation of cortical development. Early developmental history was unremarkable, with no gross or fine motor delays. He had learning difficulties and at the last visit, he was in the fifth grade but functioning on a first or second grade level academically, thus requiring special education classes with additional assistance in mathematics. At 7 years of age, he was noted to have average intellectual functioning on the KBIT‐2 and average/below average range in academic achievement on the WIAT‐III. His dysmorphisms included hypopigmented hair, high nasal bridge, micro/retrognathia, small mouth, pterygium colli, single palmar creases, fifth finger clinodactyly, persistent fetal fingertip pads, and hypoplastic nails (Figure 1). At 12 years of age his weight was 22.6 kg (−3.96 SDS), his height was 127 cm (−3.22 SDS), and his OFC was 46.5 cm (−5.3 SDS). His genetic testing work‐up included normal karyotype and chromosomal microarray analysis. ES revealed biallelic pathogenic variants in *CENPF* (c.1744G>T:p.Glu582Ter/c.7219C>T:Arg2407Ter; Figure 2). Parental testing revealed that each parent was a carrier of one of the two variants, but the unaffected brother was not tested for the variants. The p.Glu582Ter (hg19, chr:1214813425G>T) has a frequency of 4.68e‐5 5 in gnomAD, with no homozygotes; it has been reported as pathogenic for Strømme syndrome in ClinVar (ID: RCV000170523.3) with CADD score of 40, and interpreted as pathogenic according to ACMG classification (PVS1, PM2 PP3 PP5; Richards et al., 2015). The other variant p.Arg2407Ter (hg19, chr1: 214820132C>T) has a frequency of 5.26e‐5 in gnomAD, with no homozygotes; the variant has been also reported as pathogenic in ClinVar (ID: RCV000388597.1) with CADD score of 41, and interpreted as pathogenic according to ACMG classification (PVS1, PM2 PP3 PP5; Richards et al., 2015).

### 2.3 Proband 3

The proband is the second child of nonconsanguineous Caucasian parents. His older brother is in good health. He was born preterm after 35 weeks of an unremarkable pregnancy with a birth weight of 1,650 g (−2.2 SDS), length of 40 cm (−2.9 SDS), and OFC of 27.5 cm (−2.9 SDS). In the first month of life, he had feeding problems due to partial bowel obstruction, which resolved without any intervention. His motor development was within normal range. Global developmental delay was suspected from 1 year of age and became obvious later; he visited a special needs school. A WISCII showed an IQ of 48. His verbal communication was adequate. He had attention‐deficit hyperactivity disorder. He developed type 1 diabetes mellitus at the age of 6 years. On clinical evaluation at 10 years of age, his height was 137.5 cm (−0.14 SDS), his weight 34.2 kg (0.5 SDS), and OFC was 45.5 cm (−5.6 SDS). He had a sloping forehead, short palpebral fissures, and full lips (Figure 1). A brain MRI was normal. Apart from cryptorchidism requiring orchidopexy, he had no congenital anomalies and ophthalmologic evaluations ruled out malformations of the anterior segment of the eye. SNP array did not identify copy number variants. Genomic DNA from the proband and both parents were used for clinical trio ES that revealed compound heterozygous variants in *CENPF*: NM_016343.3:c.7770C>G:p.(Tyr2590Ter) maternal (hg19, chr1:214820683C>G), and NM_016343.3:c.5242G>T:p.(Glu1748Ter) paternal (hg19, chr1:214818155G>T; Figure 2). The unaffected brother was found to be heterozygous for the c.5242G>T:p.(Glu1748Ter) variant. The variants were both absent in gnomAD, were predicted to be pathogenic by SIFT and Polyphen, and their CADD scores were 37 and 36, respectively. Both variants were interpreted as pathogenic (PVS1, PM2, PP3) according to ACMG criteria (Richards et al., 2015).

### 2.4 Proband 4

The proband is the third of five children of consanguineous South Asian parents. She was born at term of gestation with a birth weight of 2700 g (−1.8 SDS). By newborn screening, she was diagnosed with hypothyroidism due to sublingual ectopic thyroid and she has been on hormonal replacement therapy with thyroxine since then. She had feeding difficulties in the neonatal period. At 1 month of age, she developed bronchiolitis and severe upper airway obstruction requiring tracheostomy that was maintained until she was 12 months of age. Her development was delayed: she walked independently by 2 years and said her first words age 4 years. She had moderate‐to‐severe learning difficulties and attended special school. Physical examination at the age 17 years, revealed a height of 150 cm (first centile), weight of 51.6 kg (24th centile), and OFC of 51 cm (−3.3 SDS). She was noted to have microcephaly with receding forehead, highly arched palate, and dental crowding (Figure 1). Upper and lower arms were thin. Her brain MRI did not reveal any cerebral abnormalities and ophthalmologic evaluations ruled out anterior segment dysgenesis of the eye. Array CGH showed normal results. Genomic DNA from the proband and both parents were used for clinical trio ES that revealed an homozygous variants in *CENPF*: NM_016343.3: c.6439C>T, p.(Arg2147Trp) (hg19, chr1:214819352C>T; Figure 2). The unaffected siblings were not tested for the variant. The variant had a frequency of 4.98e‐5s in gnomAD with no homozygotes, it is predicted as deleterious by SIFT and Polyphen with a CADD score of 25.4. The variant has been classified of uncertain significance (PM2 PP3) according to ACMG criteria (Richards et al., 2015).

## 3 MATERIALS AND METHODS

### 3.1 Exome sequencing

Case 1 The patient was enrolled in a study approved by the Ethics Committee of Federico II University Hospital in Naples, Italy (48/16). Following informed consent genomic DNA from the patient and her parents underwent ES, enriched using the SureSelect Clinical Research Exome (Agilent, Technologies, Santa Clara, CA) and sequences in the NextSeq 500 sequencing system (Illumina, San Diego, CA). A custom pipeline based on Burrows‐Wheeler Alignment tool (BWA), Genome Analysis Toolkit (GATK), and ANNOVAR (Wang et al., 2010) were used to call, annotate, filter, and prioritize variants (Musacchia et al., 2018). All candidate variants underwent to Sanger sequencing validation. BES043 was also analyzed by the same methods.

Targeted analysis of variants in cilia genes was performed on available list of cilia genes (https://nhsgms-panelapp.genomicsengland.co.uk/panels/728/v4.21).

Case 2 Genetic testing was performed in the setting of diagnostic testing. Using genomic DNA from the proband and parents, exon regions and flanking splice junctions of the genome were captured using the Agilent Clinical Research Exome kit (Agilent Technologies, Santa Clara, CA). These targeted regions were sequences simultaneously by massive parallel (NextGen) sequencing on an Illumina HiSeq 2000 sequencing system with 100 bp paired‐end reads. Bi‐directional sequence was assembled, aligned to reference gene sequences based on human genome build GRCh37/UCSC hg19 and analyzed for sequence variants using a custom‐developed analysis tool (Xome Analyzer). Sanger sequencing was used to confirm potentially pathogenic variants identified.

Case 3 ES was performed as routine diagnostics in a clinical setting. Genomic DNA was fragmented into 200–500 bp fragments by means of Adaptive Focused Acoustics (Covaris Inc, Woburn, MA) shearing according to the manufacturer's protocol. Exomes were captured using the Agilent SureSelectXT Clinical Research Exome v2 capture library kit (Agilent, Santa Clara, CA) accompanied by Illumina paired end Sequencing on the HiSeq4000 (Illumina, San Diego, CA), generating 2 × 150 bp paired end reads with at least 80× median coverage. An in‐house sequence analysis pipeline (Modular GATK‐Based Variant Calling Pipeline, MAGPIE) based on read alignment using BWA‐(MEM) and variant calling using the GATK Haplotype Caller1 and UnifiedGenotyper2 was used to align reads and call variants on the generated BAM files. Variants were subsequently annotated using the Variant Effect Predictor3. Included annotation fields were, among others, variant consequence, sift scores, polyphen scores, CADD scores, and allele frequencies in the 1,000 Genomes populations. An in‐house developed tool, additionally annotated variants using dbSNP132, gnomAD allele frequencies, and the Genome of the Netherlands (GoNL) frequencies. After annotation, variants with an allele frequency of >5% in the GoNL or in the 1000 Genomes project were excluded from further interpretation. LOVDplus (Leiden Genome Technology Center, LUMC, Leiden) was used for interpretation of variants.

Case 4 Following informed consent, the patient was enrolled in the Deciphering Developmental Disorder study, which offers trio ES for children with developmental disorders by the systematic application of the latest microarray and sequencing methods as previously described (Firth et al., 2009).

Informed consent was obtained from patients for publication at each site per local institution requirements by the authors.

### 3.2 Immunostaining of skin fibroblasts

Cells were grown on glass coverslips pretreated with poly‐lysine (Sigma‐Aldrich) to facilitate the attachment of cells in 24‐well plates and cultured in Dulbecco's Modified Eagle Medium (Gibco) supplemented with 20% FBS, 1 mM l‐glutamine, and 1% antibiotics (penicillin/streptomycin). When cells attained 90% confluence, they were cultured in serum‐free media for another 48 hr to induce ciliogenesis. Cells were fixed with ice‐cold methanol for 5 min, then permeabilized and immunostained with antibody against the ciliary component ADP‐ribosylation factor‐like GTPase 13B (ARL13B; rabbit polyclonal antibody, 17711‐1‐AP, 1:1,000 dilution; Proteintech) and the centrosome marker γ‐tubulin (mouse monoclonal antibody, T6557, 1:2,000 dilution; Sigma‐Aldrich). Donkey anti‐rabbit AlexaFluor 488 (A21206) and donkey anti‐mouse Alexa Fluor 568 (A21202; both 1:1,000 dilution; Thermo Scientific) were used as secondary antibodies. DNA was stained with Hoechst (33342, Sigma). Samples were examined under LSM700 High‐resolution confocal laser‐scanning microscopes (Zeiss) and Z‐stack images were obtained under a ×63 oil‐immersion objective at a definition of 1024 × 1024 pixels, adjusting the pinhole diameter to 1 Airy Unit for each emission channel. To perform ciliary length analysis, randomly chosen fields were scanned, using the same setting parameters and measurement was carried out on imaged cells (at least 50 cells/condition) for each of three‐independent experiments using the ImageJ plugin CiliaQ. Significance was determined with paired t‐testing. The percentage of ciliated cells was evaluated by counting the number of cilia in respect to the number of nuclei and was performed using the multi‐point tool of ImageJ (Iaconis et al., 2020).

## 4 RESULTS

**FIGURE 3:**
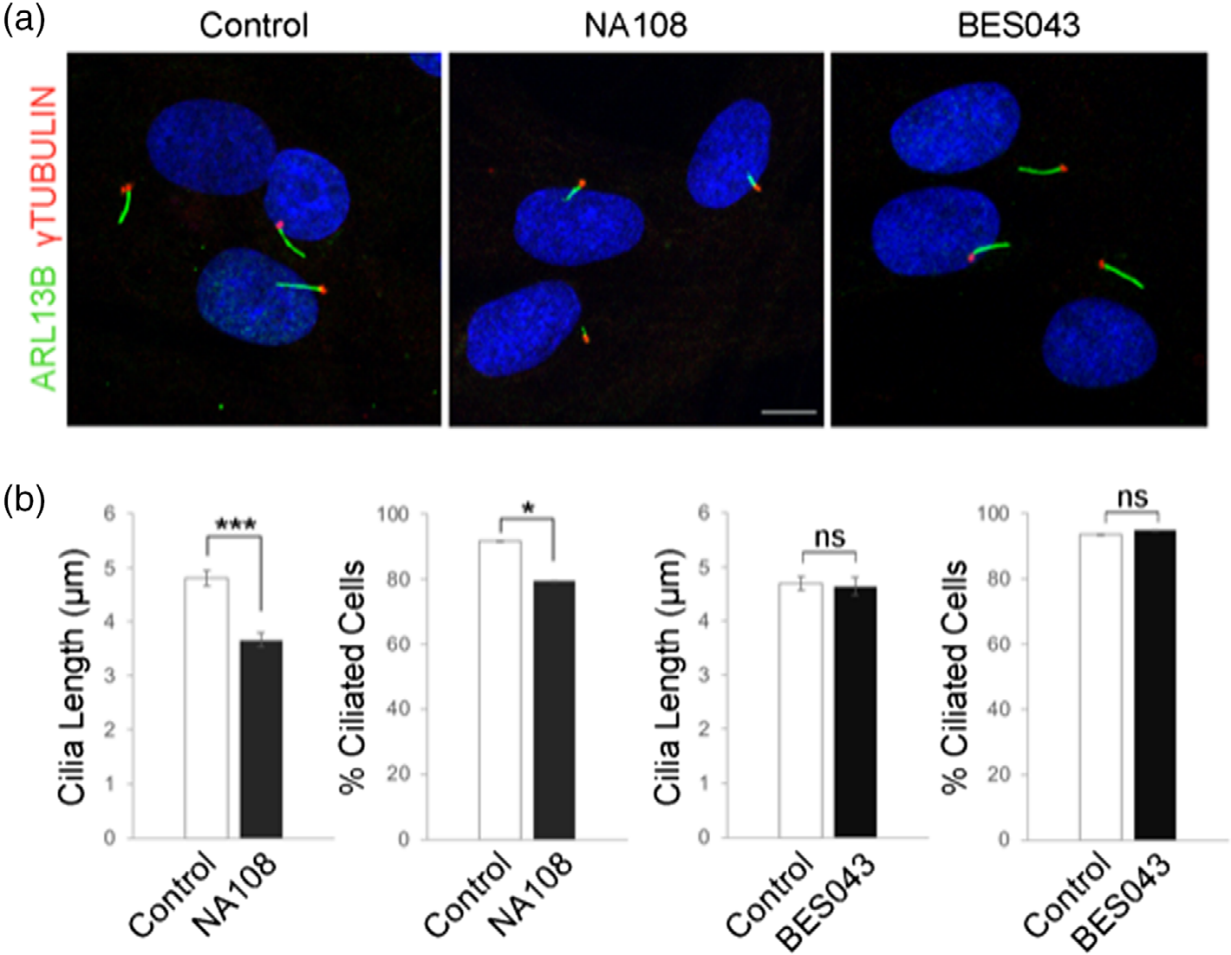
Immunofluorescence analysis of primary cilia in skin fibroblasts. (a) Representative images of fibroblast cells obtained from individuals carrying nucleotide changes in *CENPF* and a normal control after 48 hr of serum starvation. Centrioles and axonemes were immunostained for γ‐tubulin (red) and ARL13B (green), respectively. Hoechst labels nuclei (blue). Scale bar: 10 μm. (b) Graphs show the quantification of ciliary length, expressed in μm, and of cilia number, expressed as the % of ciliated cells, in NA108 and BES043 fibroblasts compared to the normal control, and ≥100 cells analyzed per sample. Data are expressed as the mean values and are representative of three independent experiments, error bars indicate the SEM. Paired Student's *t*‐test were applied. *p ≤ .05 and ***p ≤ .001. ns, not significant

Immunostaining of skin fibroblasts of proband 1 (NA108) for centrioles and axonemes with γ‐tubulin (red) and ARL13B (green), respectively, showed smaller cilia (Figure 3). The quantification of ciliary length and number confirmed the alteration in NA108 fibroblasts compared to what observed in normal control fibroblasts, and in BES043 fibroblasts that also carried two rare *CENPF* variants: p.Glu2178Lys (maternally inherited) and p.Val2387Ala (paternally inherited). BES043 fibroblasts were from a case with developmental delay but neither dysmorphic features nor microcephaly.

## 5 DISCUSSION

Centrioles are microtubule structures involved in centrosome and cilia formation (Bornens, 2012). Pathogenic variants in centrosomal and microtubule‐regulating genes have been found in disorders of neuronal migration and primary microcephaly, whereas variants in genes regulating centriole length have been detected in ciliopathy disorders presenting with heterotaxy, retinal degeneration, skeletal dysplasia, microcephaly, cerebral, and renal malformations. *CENPF* encodes a microtubule‐regulating protein involved in centromere–kinetochore complex formation, acting during both chromosome segregation around mitosis and primary cilia formation (Filges et al., 2016). Not surprisingly, the clinical presentation of Strømme syndrome due to loss of CENPF function overlaps with both ciliopathies and disorders caused by defects in centrosomal and microtubule‐regulating genes. The clinical spectrum of Strømme syndrome ranges from severe cases with early lethality to milder forms with normal survival and mild cognitive impairment.

We report four patients with biallelic *CENPF* variants and three of them were lacking the complete clinical trial of Strømme syndrome. In one of the cases (proband 1), we could provide functional evidence of a ciliary defect, supporting the pathogenicity of the *CENPF* missense variants. In contrast, another suspected case with bi‐allelic *CENPF* variants (BES043) with nonspecific developmental delay and without microcephaly did not show any morphological abnormalities of the cilia in fibroblasts. This simple assay appears to be a valid tool for confirming the pathogenic role of *CENPF* variants. However, further functional studies are needed to conclude more conclusively that missense *CENPF* variants result in loss of protein function. Moreover, validation of this assay in further cases is required.

Microcephaly was present in all cases in this series, consistent with the reported cases with biallelic *CENPF* pathogenic variants. However, few cases reported with macrocephaly associated with hydrocephalus and alobar holoprosencephaly have also been reported (Alghamdi et al., 2020).

Mice *null* for *Cenpf* showed structural kidney defects including loss of ciliary structures, tubule dilation, and disruption of glomeruli, suggesting a role for CENPF protein in renal development (Haley et al., 2019). Consistently, patients carrying *CENPF* variants have been found to have kidney malformations and end‐stage renal disease (Caridi et al., 2021; Waters et al., 2015) and most cases in our series showed genitourinary malformations including horseshoe kidney, ureteral defects, and cryptorchidism.

Pathogenic variants in genes encoding centrosomal proteins cause a variable spectrum of disorders. For example *CEP290* pathogenic variants can be responsible of multiple phenotypes attributed to cilia dysfunction including Joubert syndrome, Leber congenital amaurosis, Meckel syndrome, and Senior‐Loken syndrome (Coppieters et al., 2010). Similarly, our cohort of cases with biallelic *CENPF* variants suggests that CENPF defects might be associated with ID, microcephaly, and dysmorphic features even in the absence of the typical malformations of Strømme syndrome (i.e., anterior chamber malformation of the eye and intestinal atresia). We speculate that this wide ranges of phenotypes might be dependent on a gene‐dosage effect with biallelic LoF variants resulting in the absence of the protein product being associated with the clinical presentations that include the full spectrum of Strømme syndrome, and missense variants allowing for some degree of residual CENPF protein function being associated with phenotypes that do not include Strømme syndrome‐related internal malformations.

In conclusion, our case series expands the genetic and clinical spectrum of CENPF‐related disease.

## Data Availability

Data sharing is not applicable to this article as no new data were created or analyzed in this study.
